# Exosomes and circular RNAs: promising partners in hepatocellular carcinoma from bench to bedside

**DOI:** 10.1007/s12672-023-00672-9

**Published:** 2023-05-08

**Authors:** Mengyuan Hu, Xue Li, Zhenluo Jiang, Qing Xia, Yaoren Hu, Junming Guo, Liyun Fu

**Affiliations:** 1 School of Medicine, Ningbo University, Ningbo, 315211 China; 2 Department of Infection and Hepatology, Ningbo No. 2 Hospital, Ningbo, 315010 China; 3 Wenzhou Medical University, Wenzhou, 325035 China; 4 Department of Emergency, Ningbo No. 2 Hospital, Ningbo, 315010 China; 5 Department of Hepatopancreatobiliary Surgery, Ningbo No. 2 Hospital, Ningbo, 315010 China; 6 Key Laboratory of Diagnosis and Treatment of Digestive System Tumors of Zhejiang Province, Ningbo, 315010 China; 7 Department of Biochemistry and Molecular Biology, and Zhejiang Key Laboratory of Pathophysiology, Medical School of Ningbo University, Ningbo, 315211 China; 8 Ningbo Clinical Research Center for Digestive System Tumors, Ningbo, 315010 China

**Keywords:** Exosome, Circular RNA, Hepatocellular carcinoma, Biomarker, Mechanism, Immune therapy

## Abstract

Hepatocellular carcinoma (HCC) is characterized by high morbidity and mortality, and a low 5-year survival rate. Exploring the potential molecular mechanisms, finding diagnostic biomarkers with high sensitivity and specificity, and determining new therapeutic targets for HCC are urgently needed. Circular RNAs (circRNAs) have been found to play a key role in the occurrence and development of HCC, while exosomes play an important role in intercellular communication; thus, the combination of circRNAs and exosomes may have inestimable potential in early diagnosis and curative therapy. Previous studies have shown that exosomes can transfer circRNAs from normal or abnormal cells to surrounding or distant cells; thereafter, circRNAs influence target cells. This review summarizes the recent progress regarding the roles of exosomal circRNAs in the diagnosis, prognosis, occurrence and development and immune checkpoint inhibitor and tyrosine kinase inhibitor resistance of HCC to provide inspiration for further research.

## Introduction

Compared with the data in 2018, primary liver cancer (PLC) has risen from the fourth to the third most prevalent cause of cancer death worldwide and is still the sixth most commonly diagnosed cancer globally, with approximately 906,000 newly-diagnosed liver cancer cases and 830,000 deaths in 2020 [1–3]. Hepatocellular carcinoma (HCC) is the most common pathological type of PLC, accounting for approximately 75%-85% of cases [1]. Currently, many patients are diagnosed at advanced stages, and therapeutic methods are limited [4]. In patients with PLC between 2000 and 2014, the 5-year net survival rate was less than 20% in 92% of countries assessed [5].

Thus, efforts to explore novel early diagnostic biomarkers and develop new drugs for prolonging HCC patients’ overall survival (OS) are ongoing. For example, in the field of systemic therapy, drugs such as tyrosine kinase inhibitors (TKIs), anti-vascular endothelium growth factor (VEGF) antibody, immune-checkpoint inhibitors (ICIs), and cytotoxic T lymphocyte-associated antigen-4 (CTLA-4), etc., are continuously emerging [6]. Based on the abovementioned progress, the management of advanced HCC has been revolutionized in the past few years. For example, the FDA has approved the combination of atezolizumab, an anti-programmed death ligand (PD-L1) antibody, plus bevacizumab (anti-VEGF antibody) in the first-line setting because it is associated with a median OS of 19 months in patients with advanced HCC [6–8]. In recent years, novel ICIs including those targeting lymphocyte activation gene-3, T-cell immunoglobulin-3(TIM3), and T-cell immunoreceptor with Ig and ITIM domains, as well as agonists of the costimulatory receptors glucocorticoid-induced tumour necrosis factor receptor and inducible T-cell costimulator have entered clinical trials [9].

Exosomes are one of the main types of extracellular vesicles (EVs), and initial research has shown that exosomes are used as cleaners to discard intracellular wastes [10]. Circular RNAs (circRNAs) were once considered meaningless products in the transcription process [11]. With the progress of technology and research, researchers have increasingly realized that they have special and multiple functions [11–13]. Exosomes play an important role in tumorigenesis, immunity, metabolism, cardiovascular diseases, clinical diagnosis, drug delivery, etc. [12], while circRNAs have also been found to play a significant role in various tumours, immune escape, and many other diseases [11, 13].

Exosomes have been found to load circRNAs and transmit signals between cells [14]. Importantly, exosomal circRNAs are involved in tumorigenesis and development in cancers, including HCC, and have a prominent role in immunity in particular [15–17].

## Exosomes: A means of intercellular communication

Exosomes are characterized by a diameter of approximately 30–150 nm and a series of biomarkers, such as CD63, CD81, CD9, tumour susceptibility gene 101, Alix, and heat shock protein 70, and are surrounded by lipid bilayers [18–20]. Existing studies have shown that the formation of exosomes progress from early-sorting endosomes to late-sorting endosomes and then to multivesicular endosomes, which fuse with the plasma membrane to release exosomes [12]. Exosomes can be secreted by the majority of cell types, including tumour cells, immune cells, and normal cells [18, 21]. All cellular organisms secrete exosomes. Exosomes are extensively distributed and exist in many body fluids, such as plasma, urine, saliva, and cerebrospinal fluid [10, 22].

Exosomes have been implicated in physiological and pathological processes, such as angiogenesis [18], immune responses [23], homeostasis maintenance [24], coagulation [25], inflammation [26], cancer progression [27], and antigen presentation [28]. Nucleic acids, proteins, and metabolites delivered by exosomes into recipient cells effectively alter their biological response [12].

The versatility of exosomes has been demonstrated based on previous research; that is, they can be used as a vehicle to not only transmit various signal molecules or excrete harmful things from cells but also transport drugs, ligands, receptors, etc. [18, 24–31]. Exosomes have been demonstrated to be efficient drug conveyors with excellent biocompatibility [30]. Exosome‑based anticancer drug delivery for patients with HCC is currently developing; for example, tumour necrosis factor‑related apoptosis‑inducing ligand (TRAIL) and miR‑335 via exosome vehicles will be used in a clinical scenario in the foreseeable future [29]. Regarding antigen presentation, Cheng’s team found that macrophage-derived exosomes carrying mycobacterial antigens could protect mice against an *Mycobacterium tuberculosis* infection and have the potential to become a novel cell-free vaccine [28].

## CircRNA: An important regulatory factor in HCC

CircRNA was once considered as the noise of transcription but has attracted increasing attention in recent years. The results of several research teams have identified many important roles of multiple circRNAs in HCC [18, 23, 27].

The differential expression profile of circRNAs has been identified under different prerequisites by next-sequencing techniques or microarray analysis, and several circRNAs have been confirmed to play an important role in the early diagnosis, occurrence and development, immune escape, and drug resistance of HCC [32–34].

Li et al.[35] showed the potential diagnostic value of circ_0000098 in HCC, with an area of 0.829 under the receiver operating characteristic (ROC) curve. Further in vivo and in vitro experiments showed that circ_0000098 could play an oncogenic role by regulating mitochondrial calcium uniporter regulatory factor 1 expression by sequestering miR-383 [35]. Inhibiting the expression of circ_0000098 in HCC cells could diminish doxorubicin (DOX) resistance by decreasing the levels of intracellular ATP and the expression of the ATP-dependent drug efflux protein P-glycoprotein (P-gp) [35]. Based on this finding, DOX and a short hairpin RNA against circ_0000098 (referred to as sh-1) were encapsulated into platelets, named DOX/sh-1@PLT, which could reverse DOX resistance [35]. Wang's team confirmed that circCDYL was related to the expression of Ki-67 and alpha-fetoprotein (AFP), suggesting that circCDYL promoted the proliferation of HCC cells [36]. Yu et al. [33] found that circSMARCA5 could bind both miR-17-3p and miR-181b-5p to protect TIMP metallopeptidase inhibitor 3 from downregulation, thus inhibiting the proliferation of HCC.

Overall, a majority of current molecular mechanisms of HCC-related circRNAs are focused on competitive endogenous RNAs (ceRNAs) that compete with mRNA to bind corresponding miRNAs, thus regulating the degradation of mRNAs.

## An excellent combination of exosomes and circRNAs

CircRNA is stable because it has a deficiency of 5’ caps and 3’ tails and is covalently closed and cannot be easily degraded by exonuclease [37]. There has also been a great deal of evidence that it is abundant and functional [14, 38].

Additionally, under the protection of phospholipid bilayers, exosomes are difficult to degrade by enzymes [39], so exosomes are considered to be an excellent natural vehicle, carrying a variety of biomarkers, including circRNAs, involved in intercellular communication and affecting the tumour microenvironment (TME) and the malignant behaviour of cancer cells [39]. Rich and stable circRNAs combined with exosome protection could play a strong and stable role in tumour diagnosis and treatment and is an intriguing aera of research.

In 2015, Li’s team demonstrated for the first time the presence of abundant circRNAs in exosomes [14]. Since then on, mounting studies in different malignancies have proven this point [18, 21, 40], such as circPDK1 in serum exosomes of pancreatic cancer patients [41] and circHNRNPU in exosomes from the culture supernatant of multiple myeloma cells [13]. Their current progress is described as follows.

### Exosomal circRNAs function as diagnostic and prognostic biomarkers

Exosomal circRNAs have been reported as novel diagnostic and prognostic biomarkers for HCC [42–46].

Lin et al. [44] showed that circ_0072088 was not only upregulated in both HCC tissues and cells compared with paracancerous tissues and healthy hepatic cells but also showed high expression in HCC patient plasma exosomes. ROC curve analysis demonstrated that serum exosomal circ-0072088 had a high diagnostic value for HCC, with an area under the ROC curve (AUC) of 0.899. Kaplan–Meier curve and Cox proportional-hazards model analyses found unfavourable prognosis of HCC patients with high expression of exosomal circ_0072088.

Wang et al. [42] reported that serum exosomal hsa_circ_0028861 could discriminate HCC from chronic hepatitis B (CHB) and cirrhosis patients, with an AUC of 0.79, a sensitivity of 67.86% and a specificity of 82.69%. Furthermore, the combination of hsa_circ_0028861 and AFP exhibited better diagnostic ability (AUC = 0.86, sensitivity of 76.36%, and specificity of 86.27%).

Lyu et al. [43] demonstrated that hsa_circ_0070396 was upregulated in plasma-derived exosomes; moreover, the diagnostic power of exosomal circ_0070396 was better than that of AFP, with AUCs of 0.8574 and 0.7741, respectively, for distinguishing HCC from healthy donors and CHB patients, while AUCs of AFP were 0.781 and 0.7442. In addition, the combination of exosomal circ_0070396 and AFP showed higher diagnostic ability with AUCs of 0.9384 and 0.8499 [43].

Exosomal circANTXR1 might be a potential serum biomarker for HCC patients, with an AUC of 0.76 [45]. Hsa_circ_0004001, hsa_circ_0004123, hsa_circ_0075792, and their combination are expected to be valuable diagnostic biomarkers for HCC, with AUCs of 0.79, 0.73, 0.76 and 0.89, respectively [46].

CircTMEM45A was detected at higher levels in serum exosomes from HCC patients and exhibited diagnostic value with an AUC of 0.888 for distinguishing HCC from adjacent normal samples, and its high expression in HCC patients was related to poor OS [47].

In conclusion, many exosomal circRNAs had good diagnostic and prognostic potential, and their combination with AFP showed better diagnostic performance.

### Exosomal circRNAs participate in the proliferation of HCC

Exosomal circular RNAs are involved in the proliferation of HCC mainly by regulating the cell cycle and promoting cell growth.

Arsenite-transformed human hepatic epithelial (L-02) cells have been shown to transport circRNA_100284 into normal L-02 cells by exosomes, accelerating the cell cycle and facilitating the proliferation and malignancy of normal cells via the miR-217/EZH2 axis [48].

Adipocyte-derived exosomes can transfer circ-deubiquitylation(DB) to HCC cells, promoting HCC growth, and reducing DNA damage via the miR-34a/USP7/Cyclin A2 axis [49].

### Exosomal circRNAs participate in the metastasis of HCC

Current studies suggest that exosomal circRNAs promote HCC metastasis mainly through propagation of metastatic and invasive capacity to normal cells, epithelial-to-mesenchymal transition (EMT) and angiogenesis.

Accumulating evidence indicates that HCC cells with higher metastatic potential can endow this potential to low metastatic and non-metastatic cells through exosomes, in which circRNAs may perform a major role, thus improving the migration and invasion ability of recipient cells [50, 51] (Table 1). Wang et al. [52] found that exosomes derived from highly metastatic cells (HCCLM3) promote the migration and invasion of nonmetastatic cells (HepG2) and low metastatic cells (MHCC-97L) via the circPTGR1/miR449a/MET axis. Hepatic stellate cell (HSC)-derived exosomes could transmit circWDR25, ultimately inducing an EMT in the TME via the circWDR25/miR-4474-3p/ALOX15 axis [27].

**Table 1:** The mechanisms and functions of exosomal circRNAs in occurrence and development of HCC

Functions	Donate cells	Recipient cells	CircRNAs	Regulatory Axis	Specific functions	Year	Refs.
Prompt and Drug resistant	HCC cells (HCCLM3 and SMMC-7721 cells)	NK cells	circUHRF1	miR-449c-5p/TIM-3	Induce NK cell dysfunction; drive resistance to anti-PD1 immunotherapy	2020	Zhang et al. [77]
	Sorafenib-resistant HCC cells	HCC cells	circRNA-SORE (circRNA_104797 or circ_0087293)	Block PRP19-mediated YBX1 degradation	Sorafenib resistance in HCC	2020	Xu et al. [79]
	HCC cells (Huh-7 cells)	Macrophages	cricTMEM181	miR‑488‑3p/ CD39/ eATP–adenosine pathway	Disable CD8^+^ T cells and cause resistance to anti-PD1 immunotherapy	2021	Lu et al. [23]
	Cancer-associated fibroblast (CAFs)	HCC cells	circZFR (hsa_circ_ 0,072,088, hsa_ circRNA_103809)	Inhibit STAT3/NF-κB pathway	Promote HCC development; enhance cisplatin (DDP) resistance	2022	Zhou et al. [40]
	Lenvatinib-resistant HCC cells (LM3-LR and Hep-3B-LR)	Lenvatinib-sensitive cells (LM3-P and Hep-3B-P)	circPAK1	–	Promote HCC progression by binding to 14–3-3ζ to facilitate YAP nucleus translocation and inactivating Hippo signaling pathway	2022	Hao et al. [78]
Prompt	Hepatic stellated cell (HSC) stimulated by HCC cells (Hep3B, SMMC-7721, or HCCLM3)	HCC cells (SMMC-7721 and Hep3B)	circWDR25 (hsa_circRNA_004310)	miR-4474-3p/ALOX15; EMT	Facilitate HCC cell proliferation and invasion; promote the expression of CTLA-4 in HSCs and PD-L1 in HCC cells	2022	Liu et al. [27]
	Mature adipocytes	HCC cells (HepG2)	circ-DB (hsa_circ_0025129)	miR-34a/USP7/Cyclin A2	Promote HCC growth and reduce DNA damage	2019	Zhang et al. [49]
	HCC cells (Hep3B and MHCC97H cells)	Endothelial cells (HUVEC)	circRNA-100338	Proangiogenic activity	Enhance angiogenesis and HCC metastasis	2020	Huang et al. [18]
	HCC cells (HepG2 and SMMC-7721 cells)	Normal human hepatic cells (HL-7702 cells)	hsa_circ_0004277	Inhibit ZO-1 and promote EMT progression	Promote HCC proliferation and migration	2020	Zhu et al. [50]
	HCC cells with stronger metastatic ability (MHCC97H or LM3 cells)	Normal human hepatic cells (L02) and HCC cells with weaker metastatic ability (HepG2 cells)	circ_MMP2 (hsa_circ_0039411)	miR-136-5p/MMP2	Promote HCC metastasis	2020	Liu et al. [51]
	HCC cells (Huh7 and HepG2 cells)	T cells	circGSE1 (hsa_circ_0000722)	miR-324-5p/TGFBR1/ Smad3	Promote Treg expansion; enhance HCC proliferation, migration, and invasion	2022	Huang et al. [66]
	HCC cells (HepG2 cells)	Macrophages	hsa_circ_0074854	HuR/macrophage M2 polarization	Enhance growth, migration, invasion and EMT of HCC cells	2021	Wang et al. [74]
	Arsenite-transformed human hepatic epithelial (L-02) cells	Normal L-02 cells	circRNA_100284	miR-217/EZH2	Accelerate the cell cycle and proliferation of normal liver cells and lead to the malignant transformation of the non-transformed cells	2018	Dai et al. [48]
	HCC cells with stronger metastatic ability (LM3 cell)	HCC cells with weaker metastatic ability (HepG2 and MHCC97L cells)	circPTGR1 (hsa_circ_0008043 hsa_circ_0003731 hsa_circ_0088030)	miR449a/MET	Enhance the potential of cell migration and invasion	2019	Wang et al. [52]
	–	–	circWHSC1 (hsa_circ_0001387)	miR-142-3p/HOXA1	Facilitate HCC cell growth and metastasis	2021	Lyu et al. [84]
	HCC cells (HuH-7 cells)	HCC cells (HCCLM3 cells)	circANTXR1 (hsa_circ_0055033)	miR-532-5p/XRCC5	Prompt the proliferation, migration and invasion of HCC cells	2021	Huang et al. [45]
	HCC cells (SNU-387 and HuH-7 cells)	-	hsa_circ_0046600	miR-1258/SERBP1	Promote HCC progression	2021	Zhang et al. [85]
	-	HCC cells (SNU-387 and HuH-7 cells)	circ-ZNF652 (hsa_circ_0003258)	miR-29a-3p/GUCD1	Promote HCC cell proliferation, migration, invasion and glycolysis	2020	Li et al. [56]
	-	HCC cells (SNU-387 and HuH-7 cells)	circFBLIM1 (hsa_circ_0010090)	miR-338/LRP6	Promote HCC progression and glycolysis	2020	Lai et al. [55]
	HCC high metastatic potential cells (HuH-7 and HA22T cells)	HCC cell lines	circ_002136	miR-19a-3p/RAB1A	Promote HCC progression	2022	Yuan et al. [89]
Inhibit	macrophages	HCC cells (SMMC-7721 and HepG2 cells)	hsa_circ_0004658	miR-499b-5p/JAM3	Inhibit tumor progression and promote apoptosis in HCC cells	2022	Zhang et al. [59]
	–	–	hsa_circ_0051443	miR-331-3p/BAK1	Suppress HCC progression by promoting cell apoptosis and arresting the cell cycle	2020	Chen et al. [21]
Others	–	–	circAKT3	–	Associate with a higher risk of HCC recurrence and mortality	2020	Luo et al. [86]
	–	–	hsa‑circRNA‑G004213	miR‑513b‑5p/PRPF39	Increase the cisplatin sensitivity of HepG2 cells; an indicator for predicting the efficacy of TACE	2021	Qin et al. [32]

–, not provided; Ref., reference

Human umbilical vein endothelial cells (HUVEC) can be infected by exosomal circRNA-100338, eventually regulating vascular development and lumen formation and mediating cancer metastasis [18].

### Exosomal circRNAs participate in the metabolism of HCC

To seize nutrients, tumour cells may promote glucose, lipid and amino acid metabolism through tumour metabolic reprogramming, of which glycolysis is a hallmark feature of cancer [53]. A distinctive feature of HCC cells is enhanced glucose uptake, both in aerobic and hypoxic environments, which greatly accelerates glucose catabolism [54].

The expression of circFBLIM1 is positively correlated with glucose consumption, lactate production, ATP level, and the extracellular acidification rate and negatively correlated with the oxygen consumption rate, demonstrating that glycolysis is promoted through the circFBLIM1/miR-338/LRP6 pathway [55].

Another study showed that the level of glycolysis in HCC cells was suppressed after knocking down the expression of circ-ZNF652, via the miR-29a-3p/ GUCD1 axis [56].

### Exosomal circRNAs participate in the inhibition of HCC

Although many exosomal circRNAs are involved in the malignant behavior of HCC cells, a group of exosomal circRNAs has been found to play a role in inhibiting HCC progression (Table 1).

The Notch-recombination signal binding protein for immunoglobulin Kappa J region (RBPJ) signaling pathway participates in polarizing macrophages into the proinflammatory M1 subtype [57], and these macrophages secret proinflammatory mediators and are essential for killing tumour cells [58]. M2 subtype macrophage (tumour-associated macrophage (TAM) closely resembles the M2 subtype) are characterized by secreting anti-inflammatory mediators and could promote tumour progression and metastasis [58]. Zhang et al. [59] found that exosomes derived from RBPJ overexpressing macrophages could secrete hsa_circ_0004658 to suppress the proliferation and induce apoptosis of HCC cells by competing with junctional adhesion molecule 3 (JAM3) mRNA to target miR-499b-5p.

BCL2 antagonist/killer 1 (BAK1) is an important cell death regulator, and circ0051443 can be packaged from normal cells to HCC cells by exosomes and suppress the proliferation and migration of cancer cells via the miR-331-3p/BAK1 pathway [21]. These findings lay the foundation for further therapeutic research on exosomal circRNAs in HCC.

### Exosomal circRNAs regulates immune escape

The TME includes various kinds of immune cells, such as dendritic cells (DCs), effector T cells (Teffs), regulatory T cells (Tregs), regulatory B cells (Bregs), myeloid-derived suppressor cells (MDSCs), macrophages, and NK cells [60]. In a healthy state, these cells, together with immunomodulatory receptors and cytokines, maintain a balance, coexisting in cooperation and conflict, and can finally accomplish the recognition and presentation of antigens and eventually kill cancer cells, which is called immune surveillance [61]. However, cancer cells can disrupt the balance in various ways to evade immune surveillance and promote growth in the TME [62]. For a long time, researchers have spared no effort to develop possible treatments around the cancer-immunity cycle [61]. Fortunately, many immune-related therapies have emerged from further exploration and reflection on the molecular mechanisms of immunity. ICIs against programmed cell death protein 1 (PD-1), PD-L1, TIM-3 or CTLA-4 have made revolutionary advances in the treatment of HCC with prolonged OS and objective response rates [63].

Exosomal circRNAs have been reported to be involved in the immune escape of HCC cells and resistance to immunotherapy as well as in promoting or inhibiting tumorigenesis [23, 59].

Tregs can perturb the tumour immune microenvironment and promote immune escape by inhibiting the activation of Teffs, including CD4^+^ T cells and CD8^+^ T cells [64, 65]. A study by Huang et al. showed that exosome-delivered circGSE1 can be internalized by T cells, promoting the differentiation of CD4^+^ T cells into Tregs through the miR-324-5p/ transforming growth factor β receptor 1/Smad3 axis, and the expansion of Tregs can further promote the proliferation, migration, and invasion of HCC; thus, exosomal circGSE1 is a potential target of immunotherapy [66]. Furthermore, the authors noted that the ratio of Treg/CD8^+^ T cells may be associated with the prognosis of HCC and may reflect the antitumour effect of PD1/PDL1 inhibitors [66].

Adenosine is an important immunosuppressive factor that promotes the exhaustion of NK and T cells in the TME, while the cooperation of CD73 and CD39 can lead to the metabolism of extracellular ATP to adenosine [67–69]. Lu et al. [23] found that HCC‑derived exosomal circTMEM181 is internalized by macrophages, resulting in the upregulation of CD39 expression. Cell-specific CD39 expression in macrophages then cooperates with the CD73 specifically expressed in HCC cells to participate in the activation of the ATP-adenosine pathway, and the increase in adenosine ultimately promotes HCC progression and limites the anti-PD1 therapy response. This study indicated that blocking the interaction between macrophages and tumour cells may reverse anti-PD1 resistance.

**Fig. 1:**
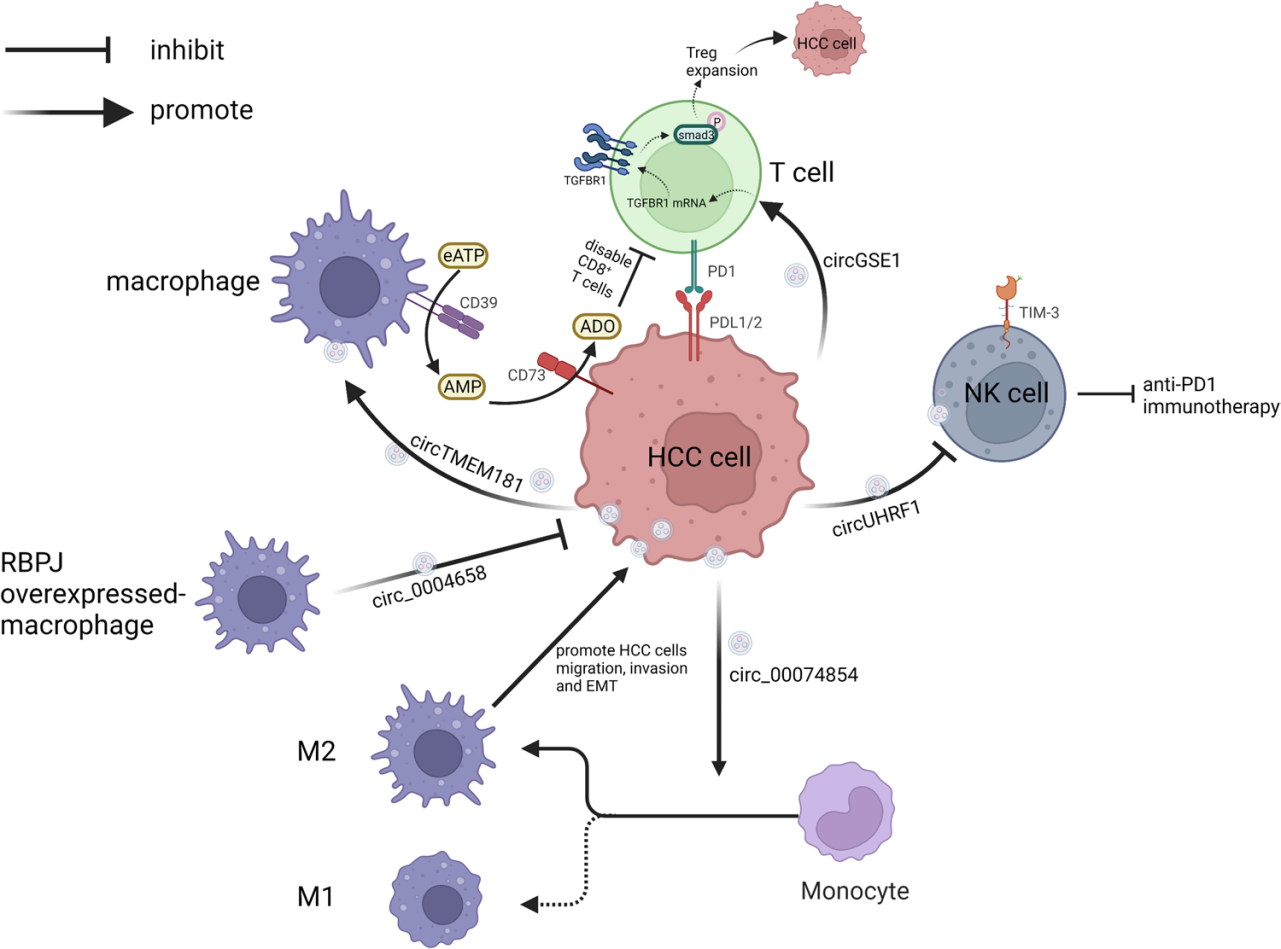
The cross-talk between HCC cells and immune cells in the HCC tumor microenvironment. a circGSE1: exosomal circGSE1 derived from HCC cells can be internalized by T cells, promoting the differentiation of CD4^+^ T cells into Tregs through the miR-324-5p/TGFBR1/Smad3 axis, and the expansion of Tregs could further promote the proliferation, migration, and invasion of HCC. b circUHRF1: exosomal circUHRF1 derived from HCC cells reduces tumor infiltration of NK cells by the miR-449c-5p/TIM-3 axis, curbing NK cell function and leading to resistance to anti-PD1 immunotherapy. ccirc_00074854: exosomal downregulated hsa_circ_0074854 derived from HCC cell inhibits the migration, invasion, and EMT in HCC cells via interacting with HuR and by suppressing macrophage M2 polarization. d circ_0004658: exosomal hsa_circ_0004658 derived from RBPJ overexpressed-macrophage inhibits HCC progression by the miR-499b-5p/JAM3. e circTMEM181: exosomal circTMEM181 promotes the activation of the spatial isolated ATP-adenosine pathway by tumor-macrophage communication, thus disabling CD8^+^ T cells and causing resistance to anti-PD1 immunotherapy

The increase in the M2/M1 macrophage ratio is another important feature of the tumor immune microenvironment [70, 71]. Previous studies have shown that a high ratio of M2 macrophages is one of the features of TAMs, which can enhance tumorigenesis [72, 73]. Lu et al. [23] found that the elevation of CD39 expression in macrophages, which occurred after the ingestion of exo-circTMEM181, could prompt polarization of M2 macrophages. Wang et al. [74] clarified that downregulated hsa_circ_0074854 reduced the stability of human antigen R (HuR) protein and decreased its protein expression and the malignant behavior of HCC cells were inhibited. They further found that HepG2 cell-derived exosomes with downregulated hsa_circ_0074854 can inhibit macrophage M2 polarization. Figure 1 shows the crosstalk between HCC cells and immune cells.

### Exosomal circRNAs induce ICI and TKI resistance in HCC

Exosomal circRNAs-mediated drug resistance has been reported in recent years [75–78], and is presented in Table 1. For example, exosomal circGSE1 and exosomal circTMEM181, which were introduced in the previous section, have potential in inducing resistance to ICIs [23, 66]. Exosomal circUHRF1, predominantly derived from HCC cells, reduces tumour infiltration of NK cells via the miR-449c-5p/TIM-3 axis, curbing NK cell function and possibly leading to resistance to anti-PD1 immunotherapy [77].

**Fig. 2:**
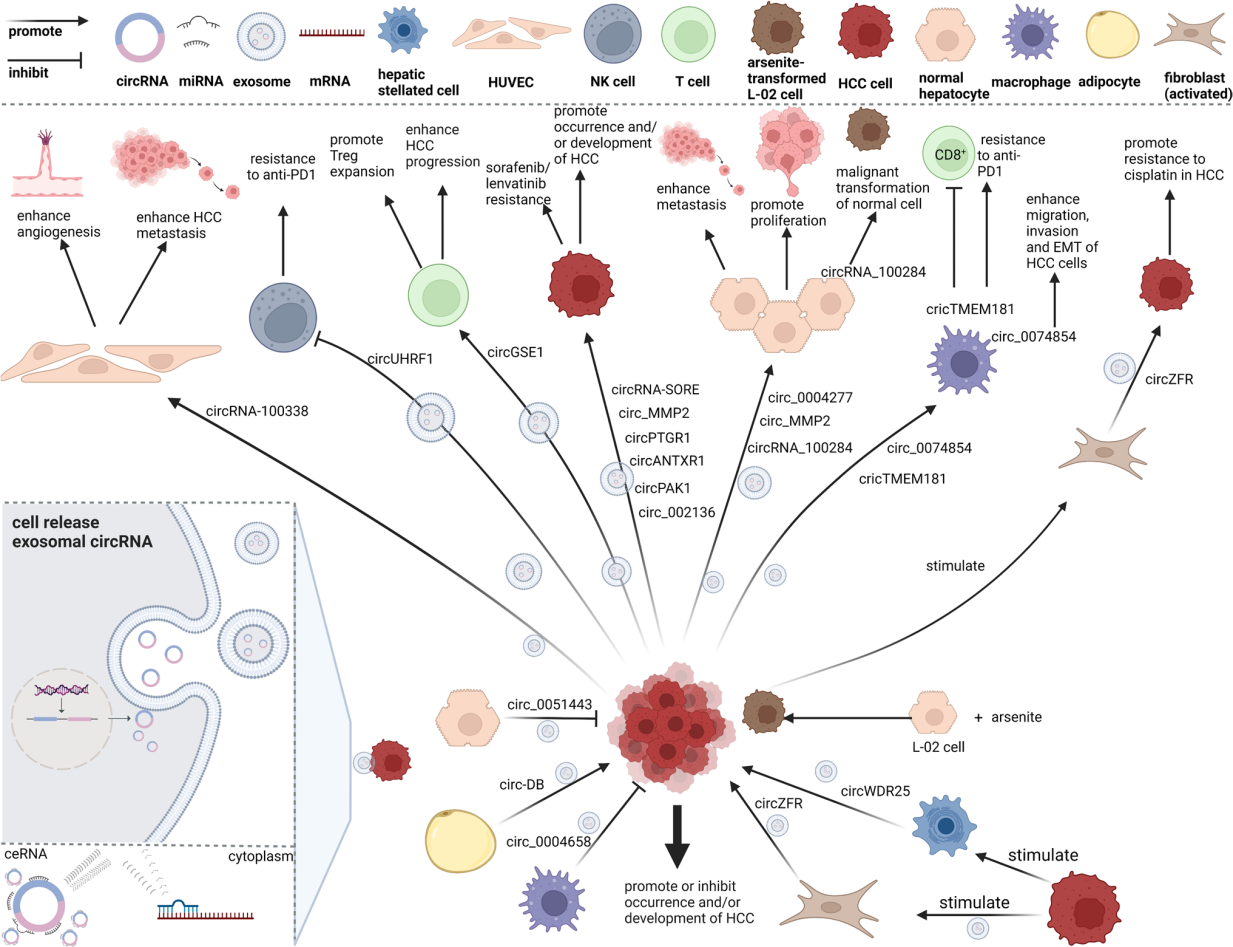
The network of circRNAs transmission mediated by exosomes in tumor microenvironment of HCC. Exosomal circRNAs can be secreted from donor cells and absorbed by recipient cells. The main underlying mechanism of HCC-related circRNAs is focused on competitive endogenous RNAs (ceRNAs) that compete with mRNA to bind corresponding miRNAs, thus regulating the degradation of mRNAs

In addition to ICIs, first line TKIs, including sorafenib, lenvatinib, and other systemic chemotherapies for patients with advanced HCC, have also been reported [6]. Xu et al. [79] found that exosomes could transport circRNA-SORE (circ-0087293) to propagate sorafenib resistance among HCC cells, and the possible resistance mechanism is that it could bind the major oncogenic protein Y-Box Binding Protein 1 and protect it against degradation. Hao et al. [78] reported that circPAK1 could be transported by exosomes from lenvatinib-resistant HCC cells to recipient parental cells and induce the resistance of recipient cells to lenvatinib. Cancer-associated fibroblasts (CAFs) can be activated by HCC cells, in a paracrine or exosomal manner, as shown in Fig. 2, thereby forming a feedback loop that further accelerates chemoresistance to cisplatin and deterioration of the tumour [80]. Zhou et al. [40] elucidated that chemoresistance to cisplatin was associated with exosomal circZFR derived from CAFs by suppressing the signal transducers and activators of the transcription/nuclear factor-kappa B pathway.

To counter the emergence of drug resistance, much research is centered on elucidating resistance mechanisms. Wei et al. [81] proposed that inhibiting the protein kinase Cα/zinc finger protein 64/CSF1 axis could reverse anti-PD1 resistance in HCC. High expression of granzyme A and coagulation factor II thrombin receptor could benefit PD-1 monoclonal antibody therapy, as demonstrated by Gao’s team [82].

## Conclusion and discussion

According to current research progress, the landscape of exosomal circRNAs in HCC has been unfolding, and an illustration of extracellular circRNA transmission mediated by exosomes is shown in Fig. 2. A growing number of studies have found that circRNAs are abundant and stable in exosomes [42–46]. This combination has already been shown to influence and expand HCC cells behaviors, namely, inducing and spreading drug resistance between malignant cells in systemic treatment and mediating immune escape [23, 66, 74–78]. However, the current series of studies on the molecular mechanisms of HCC are not comprehensive. Some studies have found that there is high expression of circRNAs in exosomes from HCC cells or patients’ blood and tissues, but the donor and/or recipient cells of exosomal circRNAs have not been further reported [32, 47, 55, 56, 83–86].Moreover, how circRNAs are sorted into exosomes and how different circRNAs play distinguishing roles in the TME are largely unknown.

Nevertheless, the combination of exosomes and circRNAs has shown potentialas diagnostic biomarkers, prognostic markers, therapeutic targets, and factors for reversing drug resistance [14, 23, 46, 47, 66, 74–78].

Ideally, a myriad of experiments will be carried out to explore new therapeutic targets, elucidate specific mechanisms of immune resistance and explore solutions to the challenges associated with HCC diagnosis and treatment [81, 82]. Outlining the exosomal circRNA landscape will provide new ideas for the management of HCC.

In the field of cancer antigen presentation, vaccines are still being attempted, and notably, exosomal and circRNA-based vaccines have emerged [87], 88]. A new generation of exosome-based therapeutic cancer vaccines has produced promising results in early clinical trials, one of which is called DEX_P&A2&N_ [88], which could be used for recruiting and activating DCs without identifying tumour antigens, thus providing personalized immunotherapy strategies for HCC patients.

In addition, research into prophylactic vaccines based on circRNAs is also progressing [87]. Nanometer materials with circRNAs have been constructed, such as chitosan/si-circPAK1 (CS/si-circPAK1) nanocomplexes, which can inhibit tumour growth and metastasis [78]. To invent an innovative exosome‑based therapy, exosomes may perform as a delivery agent of potential anticancer candidates, such as circRNA or its derivative.

Thus, the combined application of exosomes and circRNAs in inhibiting the progression of HCC as well as in immunotherapy will play an inestimable role, and one day in the future, we could see the translation of HCC-derived exosome-transmitted circRNAs in the clinic.

## Data Availability

All the data obtained and/or analyzed during the current study were available from the corresponding authors on reasonable request.

## Abbreviations

PLC: Primary liver cancer
HCC: Hepatocellular carcinoma
OS: Overall survival
TKIs: Tyrosine kinase inhibitors
VEGF: Vascular endothelium growth factor
ICIs: Immune-checkpoint inhibitors
CTLA-4: Cytotoxic T lymphocyte-associated antigen-4
ORR: Objective response rate
EVs: Extracellular vesicles
circRNAs: Circular RNAs
TRAIL: Tumor necrosis factor‑related apoptosis‑inducing ligand
DCs: Dendritic cells
ROC: Receiver operating characteristic
MCUR1: Mitochondrial calcium uniporter regulatory factor 1
DOX: Doxorubicin
P-gp: P-glycoprotein
AFP: Alpha-fetoprotein
NK: Natural killer
ceRNA: Competitive endogenous RNA
TME: Tumor microenvironment
AUC: Area under the ROC curve
CHB: Chronic hepatitis B
HSC: Hepatic stellate cell
EMT: Epithelial-to-mesenchymal transition
HUVEC: Human umbilical vein endothelial cells
TAM: Tumor-associated macrophage
JAM3: Junctional adhesion molecule 3
BAK1: BCL2 antagonist/killer 1
DCs: Dendritic cells
Teffs: Effector T cells
Tregs: Regulatory T cells
Bregs: Regulatory B cells
MDSCs: Myeloid-derived suppressor cells
PD-1: Programmed cell death protein 1
PD-L1: Programmed cell death protein–ligand 1
TIM-3: T cell immunoglobulin domain and mucin domain 3
HuR: Human antigen R
CAFs: Cancer-associated fibroblasts
